# The 9p21.3 risk of childhood acute lymphoblastic leukaemia is explained by a rare high-impact variant in *CDKN2A*

**DOI:** 10.1038/srep15065

**Published:** 2015-10-14

**Authors:** Jayaram Vijayakrishnan, Marc Henrion, Anthony V. Moorman, Bettina Fiege, Rajiv Kumar, Miguel Inacio da Silva Filho, Amy Holroyd, Rolf Koehler, Hauke Thomsen, Julie A. Irving, James M. Allan, Tracy Lightfoot, Eve Roman, Sally E. Kinsey, Eamonn Sheridan, Pamela D. Thompson, Per Hoffmann, Markus M. Nöthen, Thomas W. Mühleisen, Lewin Eisele, Claus R. Bartram, Martin Schrappe, Mel Greaves, Kari Hemminki, Christine J. Harrison, Martin Stanulla, Richard S. Houlston

**Affiliations:** 1 Division of Genetics and Epidemiology, The Institute of Cancer Research, Sutton, Surrey, United Kingdom; 2 Division of Molecular Genetic Epidemiology, German Cancer Research Centre, Heidelberg, Germany; 3 Institute of Human Genetics, University of Heidelberg, Heidelberg, Germany; 4 Northern Institute for Cancer Research, Newcastle University, Newcastle upon Tyne, United Kingdom; 5 Epidemiology and Cancer Statistics Group, Department of Health Sciences, University of York, York, United Kingdom; 6 Department of Paediatric and Adolescent Haematology and Oncology, Leeds General Infirmary, Leeds, United Kingdom; 7 Leeds Institute of Molecular Medicine, University of Leeds, Leeds, United Kingdom; 8 Paediatric and Familial Cancer, Institute of Cancer Sciences, Manchester, United Kingdom; 9 Institute of Human Genetics, University of Bonn, Bonn, Germany; 10 German Centre for Neurodegenerative Diseases, Bonn, Germany; 11 Institute for Medical Informatics, Biometry and Epidemiology, University Hospital Essen, University of Duisburg–Essen, Essen, Germany; 12 Department of Paediatric Haematology and Oncology, Hannover Medical School, Hannover, Germany; 13 General Paediatrics, University Hospital Schleswig-Holstein, Kiel, Germany; 14 Haemato-Oncology Research Unit, Division of Molecular Pathology, Institute of Cancer Research, Sutton, Surrey, United Kingdom; 15 Center for Primary Health Care Research, Lund University, Malmö, Sweden; 16 Human Genomics Research Group, Department of Biomedicine, University Hospital Basel, Basel, Switzerland; 17 Genomic Imaging Group, Institute of Neuroscience and Medicine (INM-1), Research Centre Juelich, Juelich, Germany

## Abstract

Genome-wide association studies (GWAS) have provided strong evidence for inherited predisposition to childhood acute lymphoblastic leukaemia (ALL) identifying a number of risk loci. We have previously shown common SNPs at 9p21.3 influence ALL risk. These SNP associations are generally not themselves candidates for causality, but simply act as markers for functional variants. By means of imputation of GWAS data and subsequent validation SNP genotyping totalling 2,177 ALL cases and 8,240 controls, we have shown that the 9p21.3 association can be ascribed to the rare high-impact *CDKN2A* p.Ala148Thr variant (rs3731249; Odds ratio = 2.42, *P *= 3.45 × 10^−19^). The association between rs3731249 genotype and risk was not specific to particular subtype of B-cell ALL. The rs3731249 variant is associated with predominant nuclear localisation of the *CDKN2A* transcript suggesting the functional effect of p.Ala148Thr on ALL risk may be through compromised ability to inhibit cyclin D within the cytoplasm.

Acute lymphoblastic leukaemia (ALL) is the major paediatric cancer in western countries, with B-cell precursor (BCP) ALL accounting for ~80% of cases^1^. To date the aetiology of ALL is poorly understood and while there is indirect evidence for an infective origin, no specific environmental risk factors have been identified. Evidence for inherited predisposition to ALL is provided by the increased risk seen in siblings of cases independent of the concordance in monozygotic twins, which has an *in utero* basis^2^.

By performing a genome-wide association study (GWAS) we have previously shown common SNPs at 9p21.3 annotating *CDKN2A/CDKN2B* influences ALL risk^3^. The tagSNPs associations discovered in such GWAS are however rarely themselves candidates for causality, but simply act as markers for functional variants.

To decipher the association signal at 9p21.3 we performed imputation of two GWAS datasets and subsequent replication SNP genotyping. Our analysis demonstrates that the 9p21.3 association for ALL can be ascribed to a rare high-impact haplotype at 9p21.3 which captures a *CDKN2A* exon 2 variant.

## Results

Using Haploview we defined the haplotype blocks and recombination hotspots containing the tag sentinel SNP rs3731217 (hg19 chr9:g.21984661 A > C) previously found to be associated with ALL risk at 9p21.3. rs3731217 is contained within an 174-kb LD block (at hg19 chr9:g.21,942,000–22,116,000 base pairs [bp]) and to include the possibility of long-range synthetic associations, we considered the 9q21.3 region of association to be defined by a 400kb interval (rs10965124, hg19 chr9:g.21776615 T>G to rs117009334, hg19 chr9:g.22176265 C > T). To fine map the 9p21.3 risk locus we made use of data from two previously published GWAS - (i) The German GWAS comprising 1155 B-cell ALL cases ascertained through the German Berlin-Frankfurt-Munster (BFM) group childhood ALL trials and 2125 controls from the Heinz-Nixdorf Recall (HNR) study; (ii) the UK GWAS comprising 824 B-cell cases from United Kingdom Childhood Cancer Study (UKCCS), UK MRC ALL 97 trial (http://www.thelancet.com/protocol-reviews/97PRT-14) and Northern Institute for Cancer Research; 5,200 controls from the 1958BC and UK Blood donors (http://www.cls.ioe.ac.uk/). After QC the two GWAS provided genotype data on 1,658 B-cell ALL cases (824 UKGWAS and 834 German-GWAS samples) and 7,224 controls in total. To harmonise GWAS datasets and recover untyped SNPs we made use of imputation using 1000Genomes and UK10K (http://www.uk10k.org/) as reference panels thereby allowing for the comprehensive examination of SNPs with minor allele frequency (MAF) >0.005 in the genomic region with IMPUTE2 software (https://mathgen.stats.ox.ac.uk/impute/impute).

**Figure 1:**
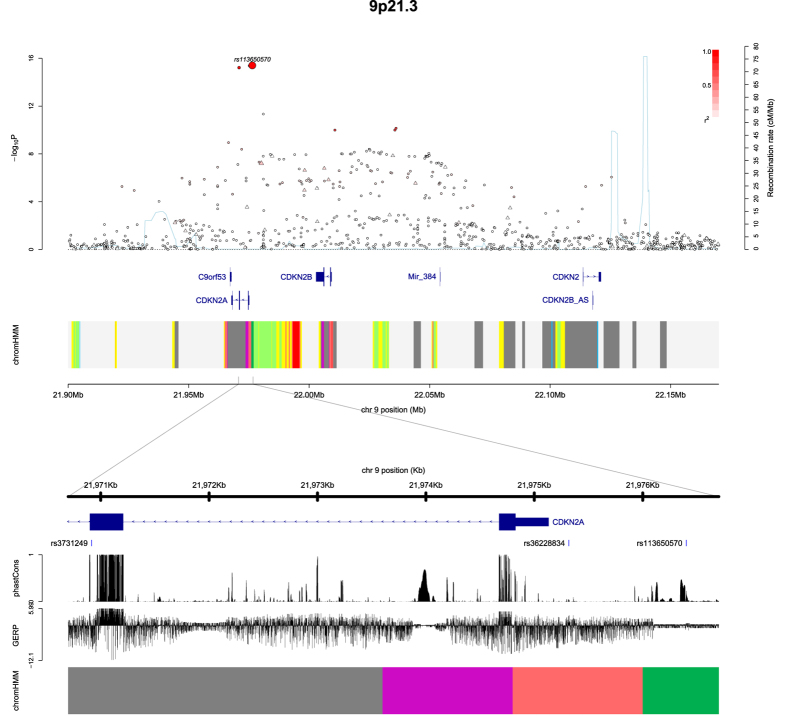
Regional plots of association results and recombination rates for the 9p21.3 risk locus. The top panel shows the association results of both genotyped (triangles) and imputed (circles) SNPs in the GWAS samples and recombination rates for rates within the two loci. For each plot, −log_10_ *P* values (y-axis) of the SNPs are shown according to their chromosomal positions (x-axis). The top SNP in the combined analysis is a large circle and is labelled by its reference SNP ID. The color intensity of each symbol reflects the extent of LD with the top genotyped SNP: white (*r*^2 ^= 0) through to dark red (*r*^2 ^= 1.0). Genetic recombination rates (cM/Mb), estimated using HapMap CEU samples, are shown with a light blue line. Physical positions are based on National Centre for Biotechnology Information build 36 of the human genome. Also shown are the relative positions of genes and transcripts mapping to each region of association. Genes have been redrawn to show the relative positions; therefore, maps are not to physical scale. The lower panel shows the gene of interest together with all transcripts of the gene showing exons and introns; observed SNP and any imputed SNPs showing a stronger association with ALL risk, chromatin state segmentation track (ChromHMM), and phastCons score values corresponding to the posterior probability associated with a phylogenetic hidden Markov model (HMM) inferring the most conserved state at a given base position. *chromHMM colour codes: *Bright Red - active promoter; Light Red - Promoter Flanking; Purple - Inactive Promoter; Orange - Candidate Strong enhancer; Yellow - Candidate Weak enhancer; Blue - Distal CTCF/Candidate Insulator; Dark Green - Transcription associated; Light Green - Low activity proximal to active states; Gray - Polycomb repressed; Light Gray - Heterochromatin/Repetitive/Copy Number Variation.*

Pooling data from each GWAS, we derived joint ORs and 95% confidence intervals (CIs) under a fixed-effects model for each SNP and the associated per-allele *P* values. The strongest association at 9p21.3 in each GWAS and in the meta-analysis were provided by the rare SNPs rs3731249 (hg19 chr9:g.21970916 G > A), rs36228834 (hg19 chr9:g.21975319 T > A) and rs113650570 (hg19 chr9:g.21976402 G > A) (MAF in cases 0.05–0.08); with combined *P*-values of 6.03 × 10^−16^, 4.15 × 10^−16^ and 3.88×10^−16^ respectively (Supplementary Table 1, Fig. 1), which was far more significant than the association provided by the sentinel GWAS SNP rs3731217 (*P*_combined _= 1.52 × 10^−8^, Supplementary Table 3). We validated rs3731249, rs113650570 and rs36228834 genotypes by sequencing 93 samples from the UK series; samples showing 100% concordance.

For replication we genotyped an additional 519 B-cell ALL cases from the UK ALL2003 trial and 1,016 population controls from the National Study of Colorectal Cancer Genetics (NSCCG) study^4^. This analysis provided increased evidence for an association between SNPs rs3731249, rs113650570 and rs36228834 and risk of ALL (Table 1). Pooling genotype data for all of three series provided unequivocal evidence for a relationship between rs3731249 (*P *= 3.45×10^−19^), rs36228834 (*P *= 4.41 × 10^−19^) and rs113650570 (*P *= 9.21 × 10^−19^) and ALL risk. There was no evidence for between study heterogeneity and combined odds ratios (OR) and 95% confidence intervals (95% CI) were 2.42 (95% CI: 1.99–2.93), 2.41 (95% CI: 1.99–2.92) and 2.39 (95% CI: 1.97–2.89) respectively (Table 1). Additionally there was no evidence that one of the series had significantly biased study findings - for rs3731249 the *P*-values for Begg’s and Egger’s tests were 1.00 and 0.35 respectively.

The SNPs rs3731249, rs113650570 and rs36228834 are in linkage disequilibrium (LD) - pairwise D’ and r^2^ metrics between rs113650570 and rs36228834, 1 and 0.99; between rs113650570 and rs3731249, 0.99 and 0.98; between rs36228834 and rs3731249, 0.99 and 0.98 respectively. Hence, while the strongest association was provided by rs3731249 the three SNPs collectively define a single risk haplotype.

To examine the possibility that the haplotype association might capture additional coding changes in *CDKN2A*, we sequenced all exons of *CDKN2A/CDKN2B* in 93 of the UK GWAS cases which were carriers of the risk haplotype. Only one additional coding change in *CDKN2A* was identified, a heterozygous deletion of G within the 3′UTR of NM 001195132 (hg19 chr9:g.21968623G>_). Collectively these data make it unlikely that the haplotype association is a consequence of association through a sequence change other than rs3731249, rs113650570 or rs36228834.

rs113650570 and rs36228834 are both non-coding SNPs which localise to intron 1 of *CDKN2A-p14ARF* (NM_058195.3). Neither of the SNPs reside within strong evolutionary conserved domains (GERP and PhastCon scores – 0.67, 0.22 and 3.44, 0.05 respectively; Fig. 1) and the genomic regions do not exhibit regulatory function as defined by histone markers or transcription factor binding. rs3731249 is responsible for the exon 2 p.Ala148Thr change in the *CDKN2A* protein transcripts p16INK4a (NP_000068; NCBI, http://www.ncbi.nlm.nih.gov/) and predicted protein transcript cdkn2a isoform X3 (XP_005251400.1; NCBI). While the genomic position to which rs3731249 maps is not evolutionarily conserved (GERP and PhastCon scores 1.06 and 0.00 respectively), protein modelling using SuSPect^5^ software implies that the p.Ala148Thr change is unlikely to be neutral and rs3731249 is predicted to be possibly damaging by PolyPhen (PolyPhen2 score = 0.49, protein accession: P42771- CD2A1_HUMAN). Direct evidence for rs3731249 being functional is provided by transfection studies^6^.

To explore the possibility that somatic loss of the wild-type allele preferentially occurs in the leukemic clones of rs3731249 carriers we examined the relationship between genotype carrier status and *CDKN2A* and *CDKN2B* deletion in the leukaemic clones of 43 UKGWAS B-cell and 519 replication series B-cell cases (Table 2). There was, however, no evidence for a relationship between rs3731249 genotype and *CDKN2A* deletion (*P *= 0.59).

The association between 10q21.2 and 10p14 and risk of BCP-ALL are highly subtype specific^5^. To investigate the relationship between variation in 9p21.3 and ALL further we conducted a stratified analysis using data on 137 ETV6-RUNX1 and 169 B-high hyperdiploid cases and rs3731249. In this analysis there was no evidence that the influence of rs3731249 genotype on ALL risk is subtype specific (Table 3).

## Discussion

Here we have provided evidence that the 9p21.3 association for risk of ALL can be ascribed to a recurrent rare high impact variant in *CDKN2A*. *CDKN2A* encodes both p16 (INK4A), a negative regulator of cyclin-dependant kinases, and p14 (ARF1), an activator of p53. *CDKN2A* and *CDKN2B* are inactivated in a number of different cancers, and mono- or biallelic deletion of *CDKN2A* are recurrent events in childhood ALL^7^. *CDKN2A* deletions are generally considered to arise as secondary events and cooperate with the initiating abnormality such as *ETV6-RUNX1* fusions to drive disease. CDKN2A/CDKN2B deletions occur in approximately 30% of childhood ALL but are often acquired at relapse where the frequency is 40%^8^,^9^,^10^.

The p.Ala148Thr variant is located towards C-terminus of the p16INK4A and is thus not within ankyrin repeats^11^ (Supplementary Figure 1) through which p16INK4A binds with CDK4. In functional studies while the variant protein has been shown to bind to CDK4 and CDK6 6 it has shown to be associated with predominant nuclear localisation of *CDKN2A* transcript. This is consistent with the functional effect of rs3731249 on ALL risk being through compromised ability to inhibit cyclin D within the cytoplasm^6^. Although a slightly higher colony count than the wild type protein has been reported in association with rs3731249, providing an indication of a subtle effect on inhibitory capabilities, the effect was not statistically significant and hence at this juncture the observation needs to with interpreted with caution.

While we found no evidence for a relationship between rs3731249 genotype and *CDKN2A* deletion this does not of course preclude the possibility that the genotype might be associated with allelic imbalances within a subclone. Indeed subclonal heterogeneity in *CDKN2A* copy number was evident in these data (Supplementary Figure 2) consistent with the published assertion that *CDKN2A* alterations are secondary events in leukaemogenesis^8^.

In conclusion, we have been able to demonstrate that the 9p21 association for ALL can be ascribed to a rare high impact variant. Given that variation at 9p21.3 has been implicated in risk of other malignancies we assessed the effect of rs3731249 on risk of other cancers with known chr9p21.3 GWAS associations but observed no association between the variant and either glioma (glioblastoma and non-GBM cancers), colorectal, lung cancer or melanoma (MelGeneDB, http://www.melgene.org) suggesting an ALL-specific role for rs3731249^12^,^13^,^14^,^15^. Further studies are required to determine the precise biological basis of the association. Finally our data also provide an important proof of principle that imputation based on large reference panels such as the UK10K can be used to detect novel, low frequency-large effect associations, thereby extending the utility of pre-existing GWAS data.

## Methods

### Ethics

Collection of samples and clinico-pathological information from subjects was undertaken with informed consent in accordance with the Declaration of Helsinki and ethical board approval. Ethical committee approval was obtained for Medical Research Council UKALL97/99 trial by individual UK treatment centres and approval for UKALL2003 was obtained from the Scottish Multi-Centre Research Ethics Committee (REC:02/10/052). Additional ethical approval was obtained under the auspices of the Childhood Leukaemia Cell Bank, the United Kingdom Childhood Cancer Study and University of Heidelberg^16^.

### Genome-wide association study

The United Kingdom (UK)-GWAS and German-GWAS have been previously reported^3^,^17^. Briefly, the UK-GWAS was based on constitutional DNA (i.e. remission samples) of 459 white BCP-ALL cases from the United Kingdom Childhood Cancer Study (UKCCS; http://www.ukccs.org/; 258 male; mean age at diagnosis 5.3 years); 342 cases from the UK Medical Research Council (MRC) ALL 97/99 (1997–2002) trial (190 male; mean age of diagnosis 5.7 years) and 23 cases from Northern Institute for Cancer Research (16 males). Genotyping was performed using Illumina Human 317K arrays (Illumina, San Diego; Available at: http://www.illumina.com). For controls we used publicly accessible data generated by the Wellcome Trust Case Control Consortium 2 (http://www.wtccc.org.uk/) from 2,699 individuals in the 1958 British Birth Cohort (Hap1.2M-Duo Custom array data) and 2,501 individuals from the UK Blood Service. The German GWAS was comprised of 1,155 cases (620 male; mean age at diagnosis 6.0 years) ascertained through the Berlin-Frankfurt-Münster (BFM) trials (1993–2004) genotyped using Illumina Human OmniExpress-12v1.0 arrays. For controls we used genotype data from 2,132 healthy individuals from the Heinz Nixdorf Recall (HNR) study; consisting of 704 individuals genotyped using Illumina-HumanOmni1-Quad_v1 and 1,428 individuals genotyped on Illumina-HumanOmniExpress-12v1.0 platform to obtain a final of 2024 individuals after quality control.

### Quality control of GWAS datasets

DNA samples with GenCall scores <0.25 at any locus were considered “no calls”. Any SNP was deemed to have failed if <95% of DNA samples generated a genotype at the locus. Cluster plots were manually inspected for SNPs considered for replication. The same quality control metrics on the German GWAS data were applied as in the UK GWAS. We removed individuals aged >16 years (n = 10); sex discrepancy issues (n = 2) and samples for whom <95% of SNPs were successfully genotyped (n = 5) (supplemental Fig. 1). We computed identity-by-state (IBS) probabilities for all pairs to search for duplicates and closely related individuals among samples (defined as IBS ≥0.80, thereby excluding first-degree relatives). For all identical pairs the sample having the highest call rate was retained, thereby eliminating 3 samples. To identify individuals who might have non-Western European ancestry, we merged our data with phase II HapMap (http://www.hapmap.org) samples (60 Western European [CEU], 60 Nigerian [YRI], 90 Japanese [JPT] and 90 Han Chinese [CHB]). For each pair of individuals, we calculated genome-wide IBS distances on markers shared between HapMap and our SNP panel, and we used these as dissimilarity measures on which to perform principal component analysis. The first 2 principal components for each individual were plotted, and 37 samples showing marked separation from the CEU cluster was excluded from the analyses. Due to the spread of the case cluster we then performed an additional principal component analysis step making use of phase III HapMap samples (111 CEU, 88 Toscans in Italy [TSI] individuals), and we removed a further 265 cases and 9 controls not present in the main cluster. We filtered out SNPs having a minor allele frequency of <1%, and a call rate of <95% in cases or controls. We also excluded SNPs showing departure from Hardy-Weinberg equilibrium at *P *< 10^−6^.

### Replication series and genotyping

The replication series comprised of 519 patients (297 male; mean age at diagnosis: 6.2 years) ascertained through the UK Medical Research Council ALL 2003 (2003–2011) trial. Immunophenotyping of diagnostic samples was undertaken using standard methods. The 1,016 controls (283 males) were ethnically-matched healthy individuals with no personal history of cancer recruited to the National Study of Colorectal Cancer Genetics (NSCCG)^4^. DNA was extracted from cell pellets using standard phenol-choloform methods. Genotyping of cases and controls was performed using competitive allele-specific polymerase chain reaction KASP chemistry (LCG Biosciences Ltd., Hertfordshire, UK). Samples having SNP call rates of <90% were excluded from the analysis. To ensure quality of genotyping in all assays, at least 2 negative controls and 1% to 2% duplicates (concordance >99.99%) were genotyped.

### Sanger sequencing

Samples were sequenced using BigDye® Terminator v3.1 Cycle Sequencing Kit (Life Technologies) using oligonucleotides mentioned in Supplementary Table 2.

### Statistical and bioinformatic analysis

Main analyses were undertaken using R (v2.6; R Core Team (2013), a language and environment for statistical computing (http://www.R-project.org/), PLINK (v1.06)^18^ and SNPTEST (v2.4.1)^19^ software. The association between each SNP and risk was assessed by the Cochran-Armitage trend test. ORs and associated 95% CIs were calculated by unconditional logistic regression. The adequacy of case-control matching and possibility of differential genotyping of cases and controls were formally evaluated using quantile-quantile plots of test statistics. The inflation factor λ was based on the 90% least significant SNPs^20^. We adjusted for possible population substructure using Eigenstrat^21^. Prediction of the untyped SNPs was carried out using IMPUTEv2 (v2.3.0) based on the data from the 1000 Genomes Project (Phase 1 integrated variant set, v3.20101123, http://www.1000genomes.org, 9 December 2013) and UK10K (ALSPAC, EGAS00001000090/EGAD00001000195, and TwinsUK, EGAS00001000108/EGAD00001000194, studies only; http://www.uk10k.org/) as reference. Imputed data were analyzed using SNPTEST v2.4.1 to account for uncertainties in SNP prediction. Association meta-analyses only included markers with info scores >0.4, imputed call rates/SNP >0.9 and MAFs >0.005. Meta-analyses were carried out with the R package meta v2.4-1, using the genotype probabilities from IMPUTEv2, where a SNP was not directly typed. To filter poorly imputed SNPs we excluded variants with information scores from SNPTEST v2.3.0 < 0.4. We calculated Cochran’s *Q* statistic to test for heterogeneity and the *I*^2^ statistic to quantify the proportion of the total variation that was caused by heterogeneity^22^. The presence of bias was formally evaluated with Begg’s adjusted rank correlation test and Egger’s regression asymmetry test^23^. Test of proportions was performed using Fishers exact test (2-sided) using STATA software (StataCorp. 2007. Stata Statistical Software: Release 10. College Station, TX: StataCorp LP).

Linkage disequilibrium (LD) metrics were calculated in PLINK^18^ using UK10K genomic data. LD blocks were defined on the basis of HapMap recombination rate, as defined by using the Oxford recombination hotspots, and on the basis of distribution of CIs^24^,^25^. Prediction of the effects of missence changes was performed using POLYPHEN. Genomic evolutionary rate profiling (GERP)^26^ and PhastCons^27^ scores were used to assess sequence conservation. SuSPect software (http://www.sbg.bio.ic.ac.uk/suspect/) was used to explore any deleterious effect of coding variant on protein function and structure^5^. The association and features plots were constructed using visPIG sofware^28^ (www.vispig.icr.ac.uk). To explore epigenetic profile of association signals, we used chromatin state segmentation data learned by computationally integrating Chip-seq data inferred from ENCODE Histone Modification data (H4K20me1, H3K9ac, H3K4me3, H3K4me2, H3K4me1, H3K36me3, H3K27me3, H3K27ac, and CTCF) and binarized using a multivariate Hidden Markov Model^29^ (http://genome.ucsc.edu/ENCODE/). We used RegulomeDB and HaploReg to examine if any of the SNPs annotate putative transcription factor binding/enhancer elements. cBioPortal (http://www.cbioportal.org) was used to explore the protein structure of the variant explored^11^.

### 9p21.3 (*CDKN2A*) copy number analysis in ALL

To study the relationship between SNP genotype and 9p21.3 monoallelic/biallelic deletion in tumours we analysed data on NCRI CCLG ALL2003 trial samples cases. Genomic copy number at 9p21.3 was assayed in diagnostic tumour samples using FISH and MLPA as previously described^7^,^8^.

## Additional Information

**How to cite this article**: Vijayakrishnan, J. *et al.* The 9p21.3 risk of childhood acute lymphoblastic leukaemia is explained by a rare high-impact variant in *CDKN2A*. *Sci. Rep.* **5**, 15065; doi: 10.1038/srep15065 (2015).

## Supplementary Material

Supplementary Information

## Figures and Tables

**Table 1:** Association between rs3731249, rs113650570 and rs36228834 genotypes and risk of developing B-cell childhood acute lymphoblastic leukaemia.

SNP	RAF^a^		OR^b^ (95% CI^c^)	*P*-value
	Cases	Controls		
rs3731249				
UK GWAS	0.05	0.03	2.48 (1.77–3.48)	1.22 × 10^−07^
German GWAS	0.08	0.03	2.46 (1.84–3.28)	9.42 × 10^−10^
Replication	0.05	0.02	2.24 (1.48–3.39)	1.40 × 10^−04^
**Combined**			**2.42 (1.99–2.93)**	**3.45 × 10**^−**19**^
				*P*_het _= 0.92, I^2 ^= 0%
rs36228834				
UK GWAS	0.05	0.03	2.52 (1.80–3.52)	6.22 × 10^−08^
German GWAS	0.08	0.03	2.44 (1.83–3.26)	1.25 × 10^−09^
Replication	0.05	0.02	2.19 (1.45–3.30)	1.90 × 10^−04^
**Combined**			**2.41 (1.99–2.92)**	**4.41 × 10**^−**19**^
				*P*_het _= 0.86, I^2 ^= 0%
rs113650570				
UK GWAS	0.05	0.03	2.52 (1.80–3.52)	6.05 × 10^−08^
German GWAS	0.08	0.03	2.44 (1.83–3.26)	1.20 × 10^−09^
Replication	0.05	0.02	2.09 (1.39–3.16)	4.10 × 10^−04^
**Combined**			**2.39 (1.97–2.89)**	**9.21 × 10**^−**19**^
				*P*_het _= 0.77, I^2 ^= 0%

^a^Risk allele frequency (RAF).

^b^Odds ratio.

^c^95% Confidence Interval.

**Table 2:** Relationship between rs3731249 risk allele and *CDKN2A* deletion in childhood B-cell ALL.

Study	rs3731249 risk allelestatus	*CDKN2A*deletion status		*P*-value
		Not deleted	Deleted	
UK GWAS	Carrier^a^	4	1	1.0
	Non-carrier^b^	30	8	
Replication	Carrier^a^	36	11	0.57
	Non-carrier^b^	378	94	
**Combined** ***P*****-value**				0.59

^a^Samples that are a carrier of rs3731249 risk allele T.

^b^samples that are non-carriers of rs3731249 risk allele T.

**Table 3:** Relationship between rs3731249 and risk of developing ALL subtypes.

ALL Subtype	rs3731249 genotypes (CC/CT/TT)	RAF^a^ in controls	*P*-value^d^
	Cases		
ETV6-RUNX1	122/14/1	0.02	0.78
Hyperdiploid	153/16/0	0.02	–

^a^RAF: risk allele frequency. ^b^OR: odds ratio and ^c^CI: confidence intervals.

^d^*P*-value from stratified analysis.
