# Supplementary material for: The 9p21.3 risk of childhood acute lymphoblastic leukaemia is explained by a rare high-impact variant in CDKN2A
Source: Sci Rep. 2015 Oct 14;5:15065. doi: 10.1038/srep15065 (PMC4604478; doi:10.1038/srep15065)
Supplement: Supplementary Information [file srep15065-s1.pdf]

SUPPLEMENTAL DATA

The 9p21.3 risk of childhood acute lymphoblastic leukaemia is explained by a rare high-impact variant in *CDKN2A*

Jayaram Vijayakrishnan[1], Marc Henrion[1], Anthony V Moorman[4], Bettina Fiege[2], Rajiv Kumar[2], Miguel Inacio da Silva Filho[2], Amy Holroyd[1], Rolf Koehler[3], Hauke Thomsen[2], Julie A. Irving[4], James M. Allan[4], Tracy Lightfoot[5], Eve Roman[5], Sally E. Kinsey[6, 7], Eamonn Sheridan[7], Pamela D Thompson[8], Per Hoffmann[9, 16], Markus M. Nöthen[9,17], Thomas W. Mühleisen[9], Lewin Eisele[11], Claus R. Bartram[3], Martin Schrappe[13], Mel Greaves[14], Kari Hemminki[2, 15], Christine J. Harrison[4], Martin Stanulla[13], Richard S. Houlston[1]

[1]Division of Genetics and Epidemiology, The Institute of Cancer Research, Sutton, Surrey, United Kingdom;

[2]Division of Molecular Genetic Epidemiology, German Cancer Research Centre, Heidelberg, Germany;

[3]Institute of Human Genetics, University of Heidelberg, Heidelberg, Germany;

[4]Northern Institute for Cancer Research, Newcastle University, Newcastle upon Tyne, United Kingdom;

[5]Epidemiology and Cancer Statistics Group, Department of Health Sciences, University of York, York, United Kingdom;

[6]Department of Paediatric and Adolescent Haematology and Oncology, Leeds General Infirmary, Leeds, United Kingdom;

[7]Leeds Institute of Molecular Medicine, University of Leeds, Leeds, United Kingdom;

[8]Paediatric and Familial Cancer, Institute of Cancer Sciences, Manchester, United Kingdom;

[9]Institute of Human Genetics, University of Bonn, Bonn, Germany;

[10]German Centre for Neurodegenerative Diseases, Bonn, Germany;

[11]Institute for Medical Informatics, Biometry and Epidemiology, University Hospital Essen, University of Duisburg–Essen, Essen, Germany;

[12]Department of Paediatric Haematology and Oncology, Hannover Medical School, Hannover, Germany;
[13]General Paediatrics, University Hospital Schleswig-Holstein, Kiel, Germany;
[14]Haemato-Oncology Research Unit, Division of Molecular Pathology, Institute of Cancer Research, Sutton, Surrey, United Kingdom; and
[15]Center for Primary Health Care Research, Lund University, Malmö, Sweden
[16]Human Genomics Research Group, Department of Biomedicine, University Hospital Basel, Basel, Switzerland
[17]Genomic Imaging Group, Institute of Neuroscience and Medicine (INM-1), Research Centre Juelich, Juelich, Germany

List of Supplemental Files:

Supplementary Table 1: Genotype counts of the top three significant SNPs from UK and German GWAS study and replication series.

Supplementary Table 2: Details of oligonucleotides used in Sanger sequencing and allele specific genotyping.

Supplementary Table 3: Results of imputation and meta-analysis of UK and German GWAS data sets showing SNPs with P-values less than 0.5X10-7 detailing genotype counts from UK and German GWAS series. a. Meta-analysis performed under fixed effects; b. Beta values showing estimates of the model parameters with allele_A as 0 and allele_B as 1 under a frequentist additive model; c. Standard error for the beta values; d.I2 is the Heterogeneity index.

Supplementary Figure 1: Protein structure of CDKN2A bearing the amino acid change p.A148T (rs3731249) showing ANKYRIN repeats and location of amino acid change highlighted in yellow.

Supplementary Figure 2: Frequency of CDKN2A deletions in ALL

	rs113650570 (GG/GA/AA)		RAF[a]	
	**Cases**	**Controls**	**Cases**	**Controls**
**UKGWAS**	749/71/4	4933/263/4	0.05	0.03
**GERGWAS**	722/100/13	1914/108/2	0.08	0.03
**Replication**	473/44/2	972/43/1	0.05	0.02
	**rs36228834 (TT/AT/AA)**		**RAF**	
	**Cases**	**Controls**	**Cases**	**Controls**
**UKGWAS**	749/71/4	4933/263/4	0.05	0.03
**GERGWAS**	722/99/13	1914/108/2	0.07	0.03
**Replication**	472/45/2	973/42/1	0.05	0.02
	**rs3731249 (CC/CT/TT)**		**RAF**	
	**Cases**	**Controls**	**Cases**	**Controls**
**UKGWAS**	750/69/4	4933/261/4	0.05	0.03
**GERGWAS**	722/101/12	1916/106/2	0.07	0.03
**Replication**	472/45/2	974/41/1	0.05	0.02

Supplementary Table 1: Genotype counts of the top three significant SNPs from the UK and German GWAS study and replication series.

a. RAF: Risk Allele Frequency

Primer_ID	Oligonucleotide sequence
cdkn2a_Com_EX01_AF	GTAGTCCCAGCACATCTTACA
cdkn2a_Com_EX01_AR	CCGAATAGTTACGGTCGGAGG
cdkn2a_Com_EX01_BF	GAAGCGCTACCTGATTCCAA
cdkn2a_Com_EX01_BR	GGTCCCTCCAGAGGATTTGA
cdkn2a_Com_EX01_CF	AGCCCCTCCTCTTTCTTCCT
cdkn2a_Com_EX01_CR	AGTGAACGCACTCAAACACG
cdkn2a_EX02_F	GCTTTGGAAGCTCTCAGGGTA
cdkn2a_EX02_R	GGGGCTCTACACAAGCTTCC
cdkn2a_EX03_F	CTCCCACACCTCCCTGGT
cdkn2a_EX03_R	GCCCATACGCAACGAGATTA
cdkn2a_EX04_AF	CGATCTTGAGACACGGCTTT
cdkn2a_EX04_AR	ATATGCCTTCCCCCACTACC
cdkn2a_EX04_BF	GCAGAAGCGGTGTTTTTCTT
cdkn2a_EX04_BR	TACATGCACGTGAAGCCATT
cdkn2a_ALT_EX01_F	TAGCCTGGGCTAGAGACGAA
cdkn2a_ALT_EX01_R	CTCAGAGCCGTTCCGAGAT
**KASPAR Primers**	
rs3731249_ALC	GAAGGTGACCAAGTTCATGCTCACCTGAGGGACCTTCCGC
rs3731249_ALT	GAAGGTCGGAGTCAACGGATTCTCACCTGAGGGACCTTCCGT
rs3731249_C1	GGGGGGCACCAGAGGCAGTA
rs36228834_ALA	GAAGGTGACCAAGTTCATGCTCCTCCGCGATACAACCTTCCA
rs36228834_ALT	GAAGGTCGGAGTCAACGGATTCCTCCGCGATACAACCTTCCT
rs36228834_C1	CTATGACACCAAACACCCCGATTCAA
rs113650570_ALA	GAAGGTGACCAAGTTCATGCTACTTTCCTTTTATGTAATAGTGATAATTCTATT
rs113650570_ALG	GAAGGTCGGAGTCAACGGATTACTTTCCTTTTATGTAATAGTGATAATTCTATC
rs113650570_C2	GTGACAGAATGAGACTCCGACTCAA

Supplementary Table 2: Details of oligonucleotides used in Sanger sequencing and allele specific genotyping.

		Alleles		[a]Meta-analysis			UK cases genotype count	UK controls genotype count			German cases genotype count	German controls genotype count		
**SNP**	**Position**	**A1**	**A2**	***P*-value**	**OR**	[d]I^2	**(AA/AB/BB)**	**(AA/AB/BB)**	**[b]UK beta**	**[c]UK SE**	**(AA/AB/BB)**	**(AA/AB/BB)**	**German beta[b]**	**German SE[c]**
**rs113650570**	**21976402**	**G**	**A**	**3.90E-16**	**2.48**	**0**	**(749/71/4)**	**(4933/263/4)**	**0.92**	**0.17**	**(722/100/13)**	**(1914/108/2)**	**0.89**	**0.15**
**rs36228834**	**21975319**	**T**	**A**	**4.20E-16**	**2.47**	**0**	**(749/71/4)**	**(4933/263/4)**	**0.92**	**0.17**	**(722/99/13)**	**(1914/108/2)**	**0.89**	**0.15**
**rs3731249**	**21970916**	**C**	**T**	**6.00E-16**	**2.47**	**0**	**(750/69/4)**	**(4935/261/4)**	**0.91**	**0.17**	**(721/101/12)**	**(1916/106/2)**	**0.9**	**0.15**
9-21980996	21980996	T	TAC	4.60E-12	2.95	0	(764/59/1)	(4982/216/2)	1.1	0.22	(771/58/5)	(1965/57/2)	1.06	0.22
rs78572337	22036055	G	A	7.10E-11	2.31	0	(766/56/2)	(4982/216/3)	0.84	0.19	(750/77/7)	(1943/80/1)	0.84	0.17
rs78266932	22035579	G	A	1.00E-10	2.31	0	(765/56/2)	(4982/215/3)	0.84	0.19	(750/78/6)	(1942/81/1)	0.83	0.17
rs36229158	22010681	G	A	1.00E-10	2.33	0	(766/56/2)	(4982/215/3)	0.84	0.19	(752/76/6)	(1942/81/1)	0.85	0.18
rs58801843	21981086	T	C	3.50E-10	0.7	63	(566/236/22)	(3285/1699/217)	-0.28	0.08	(594/223/18)	(1260/683/81)	-0.48	0.09
rs56018935	21966527	C	G	1.10E-09	1.49	0	(625/184/15)	(4233/920/48)	0.36	0.09	(609/203/22)	(1653/350/22)	0.45	0.1
9-22024966	22024966	A	G	4.00E-09	0.78	73	(303/387/135)	(1620/2585/995)	-0.18	0.05	(307/404/123)	(580/1025/419)	-0.34	0.06
rs3731246	21971989	C	G	4.10E-09	1.47	35	(620/190/14)	(4176/976/48)	0.31	0.09	(603/208/23)	(1649/353/22)	0.47	0.1
rs10811644	22025067	A	T	4.10E-09	0.78	74	(303/387/135)	(1621/2585/994)	-0.18	0.05	(308/404/122)	(580/1026/419)	-0.34	0.06
rs7035484	22025240	C	G	5.00E-09	0.78	73	(302/388/135)	(1618/2588/995)	-0.17	0.05	(307/404/122)	(581/1025/418)	-0.34	0.06
rs10965212	22023795	T	A	5.90E-09	0.79	79	(289/384/151)	(1527/2596/1076)	-0.16	0.05	(293/411/130)	(550/1022/452)	-0.34	0.06
9-22030027	22030027	C	T	6.80E-09	0.79	78	(287/387/150)	(1523/2595/1082)	-0.16	0.05	(293/410/131)	(549/1022/453)	-0.34	0.06
rs7044859	22018781	T	A	7.20E-09	0.79	81	(302/386/136)	(1640/2579/981)	-0.16	0.05	(311/407/116)	(590/1023/411)	-0.35	0.06
rs1591136	22026834	G	C	7.30E-09	0.79	77	(288/385/151)	(1523/2596/1080)	-0.16	0.05	(292/411/131)	(549/1022/454)	-0.34	0.06
rs7049105	22028801	A	G	8.00E-09	0.79	76	(287/387/150)	(1523/2597/1080)	-0.16	0.05	(291/411/132)	(547/1023/454)	-0.33	0.06
rs10811645	22049656	G	A	9.00E-09	0.79	74	(284/386/154)	(1499/2587/1115)	-0.17	0.05	(291/414/128)	(556/1020/448)	-0.33	0.06
rs10811640	22013411	G	T	9.60E-09	0.79	81	(301/387/136)	(1637/2580/983)	-0.16	0.05	(310/407/117)	(592/1018/413)	-0.35	0.06
9-22013805	22013805	C	T	1.00E-08	0.79	81	(301/387/136)	(1640/2580/981)	-0.16	0.05	(311/406/117)	(593/1019/413)	-0.35	0.06
9-22017550	22017550	A	G	1.00E-08	0.79	82	(300/388/136)	(1638/2578/984)	-0.15	0.05	(311/406/117)	(590/1021/413)	-0.35	0.06
9-22029445	22029445	G	A	1.00E-08	0.79	80	(287/387/150)	(1533/2592/1075)	-0.16	0.05	(296/407/131)	(551/1021/452)	-0.34	0.06
9-22017101	22017101	G	T	1.00E-08	0.79	82	(299/390/135)	(1641/2573/986)	-0.16	0.05	(313/403/118)	(591/1023/410)	-0.35	0.06
rs3731222	21983914	T	C	1.10E-08	0.71	0	(659/149/16)	(3784/1306/110)	-0.3	0.08	(686/140/9)	(1521/468/35)	-0.41	0.1
9-22032119	22032119	A	G	1.10E-08	0.79	79	(287/386/151)	(1529/2592/1079)	-0.16	0.05	(296/408/131)	(552/1021/451)	-0.34	0.06
9-21993964	21993964	T	C	1.10E-08	0.71	0	(659/150/15)	(3779/1314/107)	-0.32	0.08	(683/142/9)	(1525/463/35)	-0.38	0.1
9-21998660	21998660	G	A	1.20E-08	0.79	79	(302/390/131)	(1655/2580/965)	-0.16	0.06	(327/391/117)	(616/1019/389)	-0.35	0.07
rs150992041	22049891	TA	T	1.30E-08	0.79	76	(284/386/154)	(1508/2586/1106)	-0.16	0.05	(298/408/128)	(562/1015/447)	-0.33	0.06
9-22049555	22049555	C	T	1.40E-08	0.79	76	(284/386/154)	(1508/2585/1107)	-0.16	0.05	(297/408/129)	(562/1015/447)	-0.33	0.06

rs2151280	22034719	G	A	1.40E-08	0.79	82	(286/387/151)	(1534/2596/1070)	-0.15	0.05	(299/404/131)	(552/1023/449)	-0.35	0.06
9-22048391	22048391	C	T	1.50E-08	0.79	76	(284/386/154)	(1509/2587/1104)	-0.16	0.05	(297/408/129)	(562/1015/447)	-0.33	0.06
**rs3731217**	**21984661**	**A**	**C**	**1.50E-08**	**0.71**	**0**	(659/149/16)	(3778/1311/111)	**-0.31**	**0.08**	(685/140/9)	(1523/468/33)	**-0.39**	**0.1**
rs13297747	21980941	G	C	1.60E-08	0.71	0	(655/153/16)	(3765/1323/113)	-0.3	0.08	(681/145/8)	(1516/472/36)	-0.4	0.1
rs7028570	22048683	G	A	1.60E-08	0.79	75	(284/386/154)	(1508/2585/1107)	-0.16	0.05	(297/408/129)	(564/1014/447)	-0.33	0.06
rs1360590	22041443	T	C	1.60E-08	0.79	80	(284/387/153)	(1520/2595/1085)	-0.15	0.05	(295/408/131)	(550/1024/449)	-0.34	0.06
rs10757265	22048859	T	C	1.60E-08	0.79	77	(284/384/156)	(1509/2581/1110)	-0.16	0.05	(296/409/129)	(561/1016/448)	-0.33	0.06
9-22043819	22043819	T	C	1.60E-08	0.79	80	(285/387/152)	(1528/2587/1085)	-0.15	0.05	(295/412/127)	(557/1020/447)	-0.34	0.06
rs10738605	22049130	C	G	1.70E-08	0.79	75	(284/386/154)	(1509/2584/1107)	-0.16	0.05	(296/409/129)	(562/1015/447)	-0.33	0.06
rs10120806	22047945	T	C	2.40E-08	0.8	78	(283/386/155)	(1512/2580/1109)	-0.16	0.05	(292/414/128)	(557/1018/449)	-0.33	0.06
rs3731198	21989477	T	C	2.80E-08	0.72	0	(658/150/16)	(3777/1313/110)	-0.3	0.08	(685/139/10)	(1526/465/33)	-0.38	0.1
rs3731204	21987584	T	C	2.80E-08	0.72	0	(657/151/16)	(3774/1315/111)	-0.3	0.08	(685/140/9)	(1525/466/33)	-0.39	0.1
rs490005	22020493	G	A	3.50E-08	1.26	58	(163/391/270)	(1167/2620/1413)	0.18	0.06	(146/427/261)	(486/1022/516)	0.31	0.07
9-21979204	21979204	G	A	4.90E-08	0.71	10	(17/197/610)	(62/1037/4101)	-0.28	0.08	(25/218/591)	(30/385/1609)	-0.41	0.09
9-22050613	22050613	A	G	4.90E-08	0.8	73	(279/390/155)	(1497/2588/1115)	-0.16	0.05	(286/416/131)	(558/1011/454)	-0.32	0.06
9-22053687	22053687	A	G	5.70E-08	0.8	70	(277/392/155)	(1483/2596/1122)	-0.16	0.05	(283/419/133)	(557/1010/457)	-0.31	0.06
9-22053709	22053709	A	G	5.80E-08	0.8	71	(277/391/155)	(1489/2590/1122)	-0.16	0.05	(283/419/131)	(558/1011/455)	-0.31	0.06
rs10757267	22052810	G	C	6.00E-08	0.8	71	(277/391/155)	(1489/2590/1121)	-0.16	0.05	(284/419/131)	(559/1010/455)	-0.31	0.06
rs2811709	21980151	A	G	6.10E-08	0.71	25	(17/196/611)	(62/1037/4101)	-0.27	0.08	(25/217/592)	(30/383/1611)	-0.42	0.09
9-22055048	22055048	A	G	6.30E-08	0.8	72	(277/392/155)	(1487/2590/1123)	-0.16	0.05	(284/419/131)	(558/1012/454)	-0.31	0.06
rs567453	22021737	G	C	6.60E-08	1.25	63	(180/389/255)	(1257/2628/1316)	0.17	0.05	(161/423/249)	(522/1021/482)	0.31	0.06

Supplementary Table 3: Results of imputation and meta-analysis of UK and German GWAS data sets showing SNPs with *P*-values less than $0.5X10^{-7}$ detailing genotype counts from UK and German GWAS series. a. Meta-analysis performed under fixed effects; b. Beta values showing estimates of the model parameters with allele_A as 0 and allele_B as 1 under a frequentist additive model; c. Standard error for the beta values; d.I^2 is the Heterogeneity index.

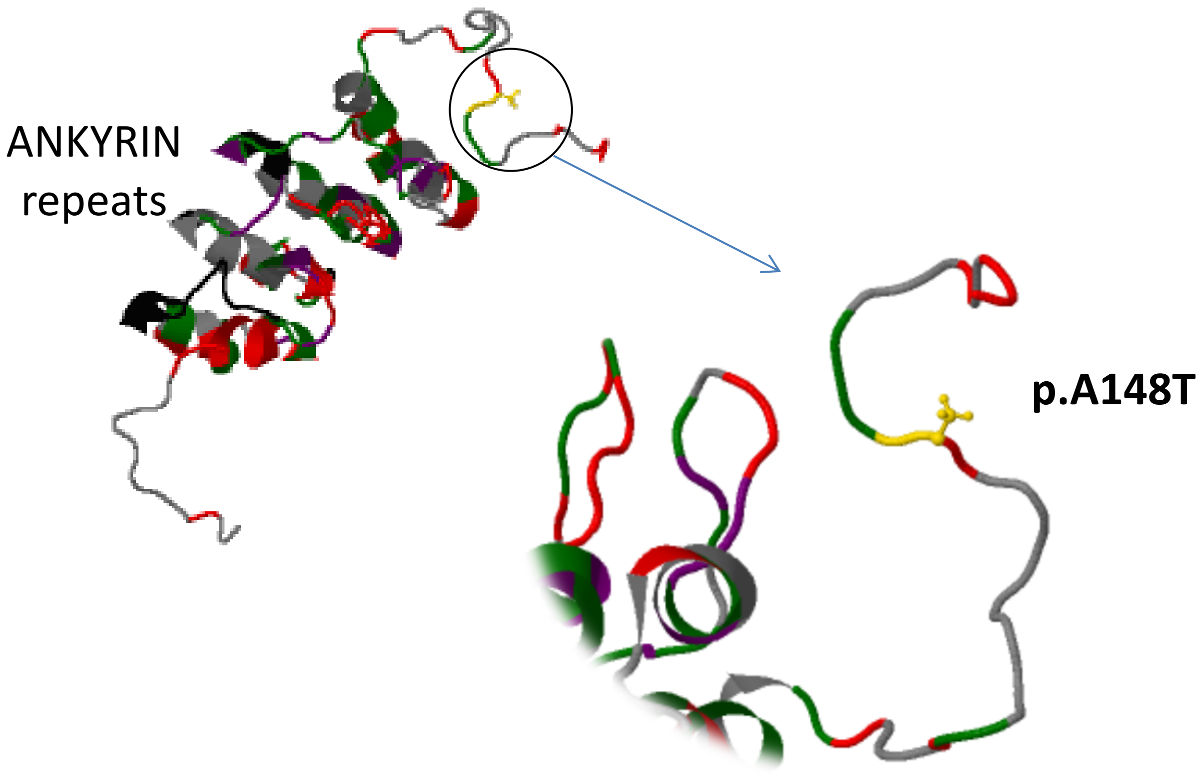

Supplementary Figure 1: Protein structure of *CDKN2A* bearing the amino acid change p.A148T (rs3731249) showing ANKYRIN repeats and location of amino acid change highlighted in yellow.

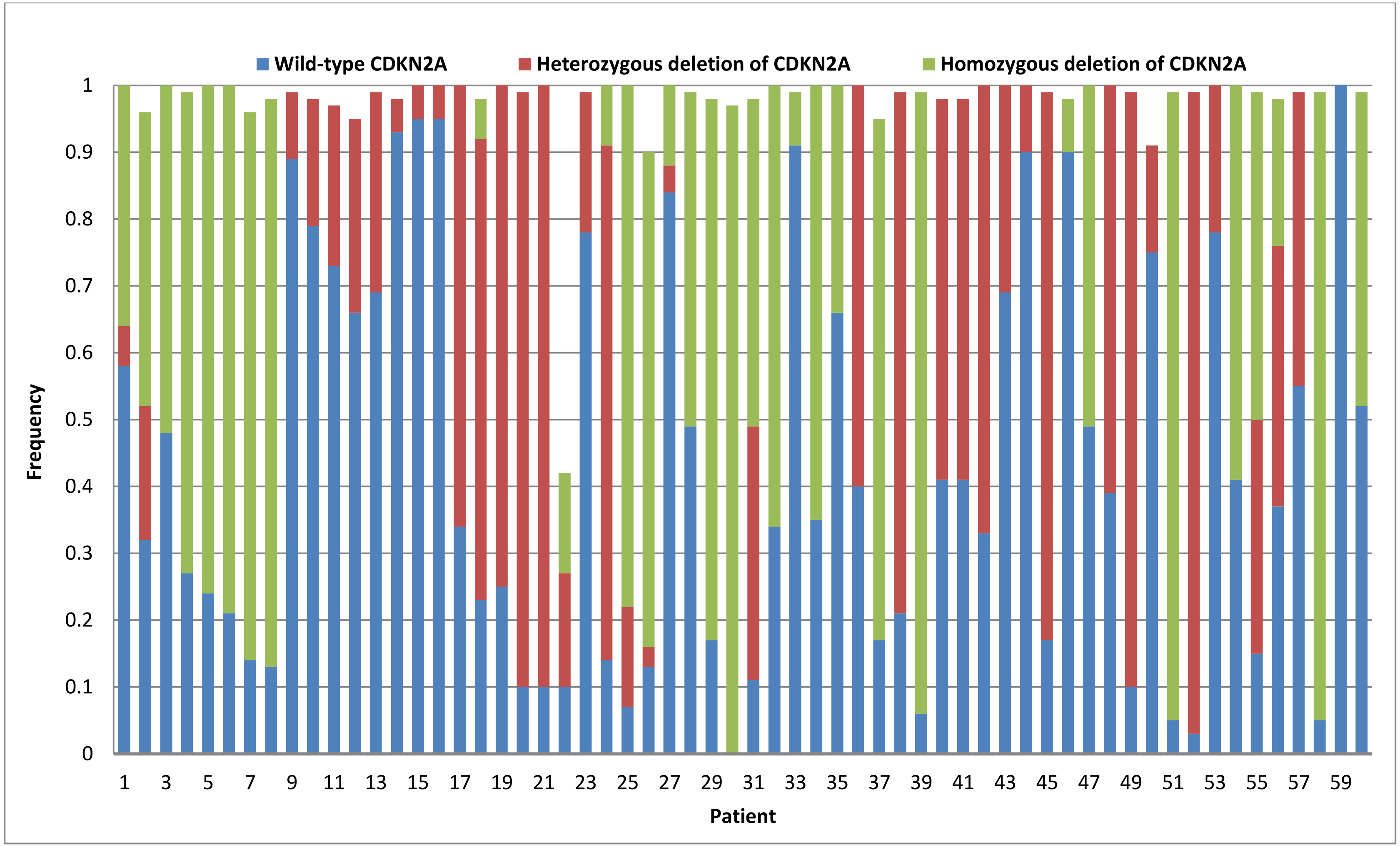

Supplementary Figure 2: Frequency of *CDKN2A* deletions in ALL
